# Artificial intelligence enhanced contemporary pulmonary hypertension care

**DOI:** 10.1016/j.ahjo.2025.100673

**Published:** 2025-11-12

**Authors:** Pyi Naing, Gregory M. Scalia, Dale Murdoch, Isuru Ranasinghe, Doug L. Forrester, Geoff Strange, David Playford

**Affiliations:** a The Prince Charles Hospital, Australia; b University of Notre Dame, Australia; c University of Queensland, Australia; d Caboolture Hospital, Australia; e University of Western Australia, Australia; f Sir Charles Gairdner Hospital, Australia; g Mandalay Heart Care, Brisbane, Australia

**Keywords:** Pulmonary hypertension, Echocardiography, Artificial Intelligence

## Abstract

All-cause pulmonary hypertension (PH) is associated with increased mortality and an enormous public health concern. However, given its complexity, multiple potential etiologies and inherent diagnostic challenges, PH diagnosis may be delayed or missed entirely. Artificial Intelligence (AI) shows potential to provide a simple but multifaceted, personalized approach for early identification of PH. AI-assisted patient triage may help highlight individuals requiring further investigation. Echocardiography may improve the identification of PH due to left heart disease and PH from other causes using a combination of AI systems such as image guidance, auto-measurement, deep phenotyping and smart reporting. Multi-level AI integrating clinical and echocardiographic data has the potential to democratize access to medical care and assist in selecting those most at risk for thorough evaluation at expert centers. In this state-of-the-art review, we discuss how new technology including AI can assist in improving the diagnosis and management of PH.

## 1 Introduction

**Fig. 1:**
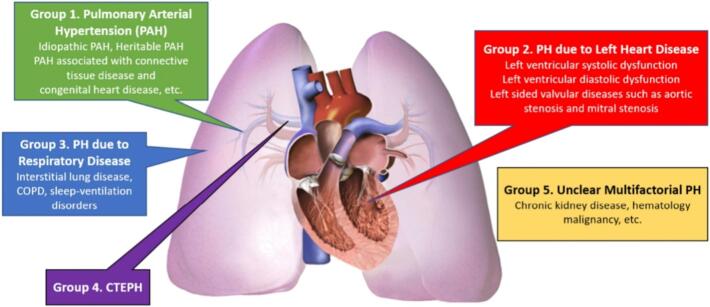
Simple illustration of pulmonary hypertension clinical classifications (image reproduced with permission from authors; Intern Med J. 2023 Jan; 53(1): 12–20. doi: https://doi.org/10.1111/imj.15860). PAH, pulmonary arterial hypertension; COPD, chronic obstructive pulmonary disease; CTEPH, chronic thromboembolic pulmonary hypertension.

Pulmonary hypertension (PH) is an abnormal hemodynamic condition due to complex interactions of the heart, lungs, and pulmonary vasculature. It is not a single disease but rather a condition which can manifest in the setting of one or more predisposing exposures or disease processes. The commonest form of PH is due to left heart disease (World Health Organization Group 2 PH, Fig. 1) and is typically first diagnosed using echocardiography. Irrespective of the underlying cause, once established, and especially if untreated, PH may lead to right heart failure and death. There is therefore a strong clinical and societal imperative to identify PH early, and institute appropriate disease-modifying therapies to improve symptoms and quality of life, and to prevent recurrent hospitalization and premature death. Symptomatic exertional dyspnea is the most common presenting symptom of PH [1], a consistent characteristic that may be extracted using clinical automation systems. Personalized tailored care may be possible for PH patients with the help of AI enhanced diagnostics and therapeutics.

There is a strong imperative to apply new and existing technologies to improve identification and risk stratification of PH, particularly since recent evidence suggests a higher burden of disease in individuals living in remote and disadvantaged regions [2], despite these same people having the least access to the current tertiary-centric PH model of care [1]. With a large global burden of disease (1 % of world's population is now estimated to have PH) [1] we propose application of new technologies that may prove cost-effective, equitable, efficient and widely applicable. Artificial intelligence (AI) has the potential to be applied along multiple points in the care pathway, and thereby offer a more efficient and valuable method of PH triage than current models [3]. Ongoing research, education and training of future PH clinicians will be required to demonstrate value across the entire care pathway [3].

We reviewed literature published between 2015 and 2025 across major databases, including Scopus and PubMed, as well as relevant conference proceedings. Our focus was on studies applying artificial intelligence to pulmonary hypertension, using keywords such as “pulmonary hypertension,” “artificial intelligence,” “machine learning,” “echocardiography,” “risk stratification,” “prognosis,” “classification,” and “diagnosis.” Our aim was to highlight clinically relevant and methodologically transparent work, rather than to conduct a systematic review.

## 2 Challenges with current model of care for pulmonary hypertension patients

**Fig. 2:**
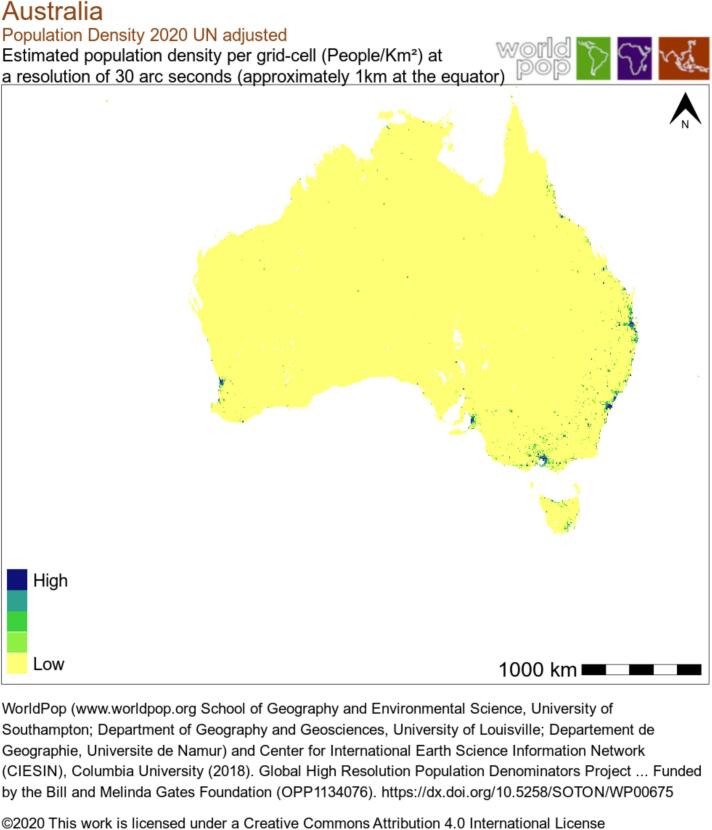
Population density map of Australia showing dispersed population resulting in health care delivery challenges.

Current guidelines suggest focusing on two main goals during PH diagnostic workup: Raising early suspicion of PH and identifying underlying etiology [1]. Despite their undisputed importance, both goals are difficult to achieve with current practice in remote, rural and disadvantaged populations. Clinical assessment and comprehensive tests such as electrocardiogram (ECG), chest radiograph, pulmonary function test, laboratory blood evaluation, chest computed tomography (CT), pulmonary ventilation and perfusion scan, echocardiography, and right heart catheterization (RHC) are all critical to thorough evaluation for possible PH. Given the estimated global burden of PH (which itself is likely an underestimate), across many nations and geographics, it is not feasible to offer the complete armamentarium of testing to all patients. As an example, Australia has a population density of 3.4 individuals per square kilometers in June 2022, and in remote areas such as Northern Territory, this may be as low as 0.2 individuals per square kilometers (Fig. 2) [4]. This dispersed population makes comprehensive PH care delivery more challenging.

Although right heart catheterization (RHC) is the gold standard for diagnosis of PH, defined as a mean pulmonary artery pressure (mPAP) > 20 mmHg [1], it is not readily available, particularly outside of tertiary centers, and is an invasive procedure with associated potential risks. RHC is particularly indicated to help define underlying pathophysiology when PAH is suspected, and usually a requirement to receive Government funded PAH therapies [5]. Moreover, the PH guidelines and most PH centers focus predominantly on risk stratification and treatment of PAH (Group 1 PH), which is rare [1] and only accounts for 2.7 to 4 % of all causes of PH [2,6]. Expert PH centers can also offer effective treatment for patient with chronic thromboembolic PH (CTEPH or WHO group 4 PH). Recommendations for other forms of PH predominantly surround treatment of the underlying condition such as heart and/or lung disease, most of which can be identified using simple investigations such as echocardiography, spirometry and chest radiology. Exertional dyspnea, the single most common presenting symptom of PH [1], is also one of the most frequent presenting symptoms to primary care [7]. Despite this symptom being nonspecific, when judicious questions are combined with basic clinical data, dyspnea may help guide further investigation.

As such, we previously developed a simpler approach to the initial clinical investigation of PH, starting with the identification of patients with unexplained dyspnea, as shown in Fig. 4. This simple clinical diagnostic algorithm is not intended to identify every underlying cause of PH as described in the European Society of Cardiology and European Respiratory Society (ESC/ERS) 2022 PH Guidelines [1], but rather to identify patients who need further investigation and help to triage those needing referral to an expert center. We propose using estimated right ventricular systolic pressure (eRVSP) cutoff >30 mmHg to suspect PH, in contrast to tricuspid regurgitation velocity > 2.8 m/s^1^, as eRVSP is more frequently and routinely reported than TRV in most echocardiogram reports. In addition, the threshold at which mortality rises corresponds to eRVSP >30 mmHg in multiple previous reports [[8], [9], [10], [11], [12], [13], [14]]. This consideration is especially important to achieve democratization of PH care through an easy-to-implement algorithm for the general medical community. Further improvement of this approach may be achieved through modern technologies such as artificial intelligence (AI).

## 3 Important therapeutic advances for pulmonary arterial hypertension

Improved understanding of pathophysiology of PAH has led to several important advances in PAH therapies and associated morbidity and mortality benefits. These agents target specific pathways responsible for pulmonary vasoconstriction, smooth muscle cell proliferation and vascular remodeling, and include Endothelin Receptor Antagonists (ERA), Phosphodiesterase 5 inhibitors (PDE5i), guanylate cyclase stimulators, prostacyclin analogues and prostacyclin receptor agonists [1,5,15]. These therapies are most beneficial in PAH and may be harmful in patients with PH due to left heart diseases (PH-LHD) and PH due to respiratory diseases (PH-RD). In Fig. 4 we outline a simple diagnostic algorithm for patients presenting with dyspnea, with echocardiography as the central investigation for PH-LHD which accounts for approximately 2/3 of all causes of PH [1,2,6,16], serving as an initial triage to distinguish left heart disease and possible PAH [17,18]. In patients with evidence for PH but no evidence of left sided valvular heart disease or systolic or diastolic left ventricular dysfunction, further evaluation in an expert center may be appropriate.

**Fig. 3:**
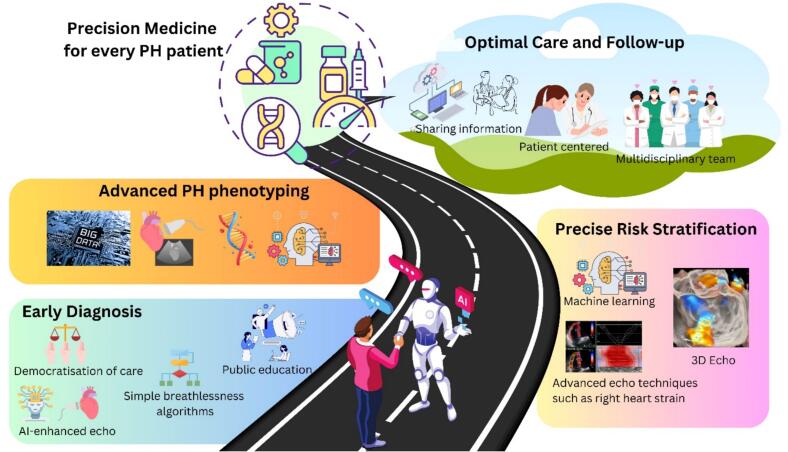
(Central Illustration). Roadmap to Precision Medicine for Pulmonary Hypertension patients.

**Fig. 4:**
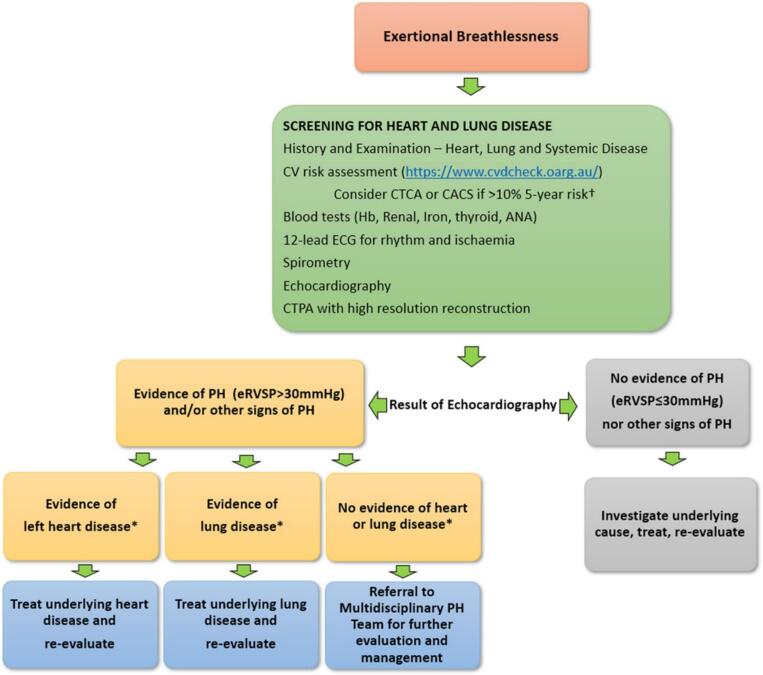
Proposed simple diagnostic algorithm for detection of pulmonary hypertension in breathless patients in remote regions. ^†^In patients with cardiovascular risk factors especially if no cause was found after the initial investigations. * The term “No evidence of heart or lung disease” refers to whether the degree of PH is proportional to the amount of left heart or lung disease observed. CTCA, CT coronary angiogram; CTPA, computed tomography pulmonary angiogram; CV, cardiovascular; ECG, electrocardiogram; eRVSP, estimated right ventricular systolic pressure; FBE, full blood examination;(image reproduced with permission from authors; Intern Med J. 2023 Jan; 53(1): 12–20. DOI: https://doi.org/10.1111/imj.15860).

In the era of advanced therapeutics for PAH, the need for precise and early diagnosis has become a powerful clinical imperative. Despite the simplified flow chart described in Fig. 4, multiple challenges remain. The process remains fully manual, relying on clinical expertise with skilled history-taking, thorough clinical examination, and the use of diagnostic tests that depend on a high skill level of the operator. The results of the testing require interpretation from clinicians with expertise and knowledge of PH. To optimize diagnostic opportunities in communities without ready access to these skill sets, further innovation is required. Access to automated techniques for echocardiographic image capture, image interpretation, measurement and phenotype detection may have a role in assisting clinicians to achieve these goals. Following is a proposed roadmap that may assist our quest to reach “precision medicine” for every patient with PH (Fig. 3, Central Illustration).

## 4 Roadmap to AI-assisted precision medicine

### 4.1 Early and accurate diagnosis through smarter triage and AI enhanced echocardiography

The data contained in electronic medical records (EMR) includes diagnostic codes and major health issues that can be captured to provide a “breathlessness triage” focused on common conditions such as coronary heart disease, hypertension, diabetes, kidney or lung diseases. Along with previously acquired laboratory, radiology, spirometry, and cardiac investigations (such as electrocardiography and cardiac imaging data), the prior medical history may be pre-filled into the model proposed in Fig. 4 [19] and may assist in the triage of patients to undergo further screening for PH. As a modality, echocardiography is well suited to identification of diseases of the left heart, in particular left ventricular systolic and diastolic dysfunction, chamber dilatation, mitral and aortic valve disease. Thus, a simplified echocardiographic approach with the goal of identifying left heart disease, and “not” left heart disease PH may offer significant benefits.

#### 4.1.1 Differentiating left heart vs non-left heart PH

**Fig. 5:**
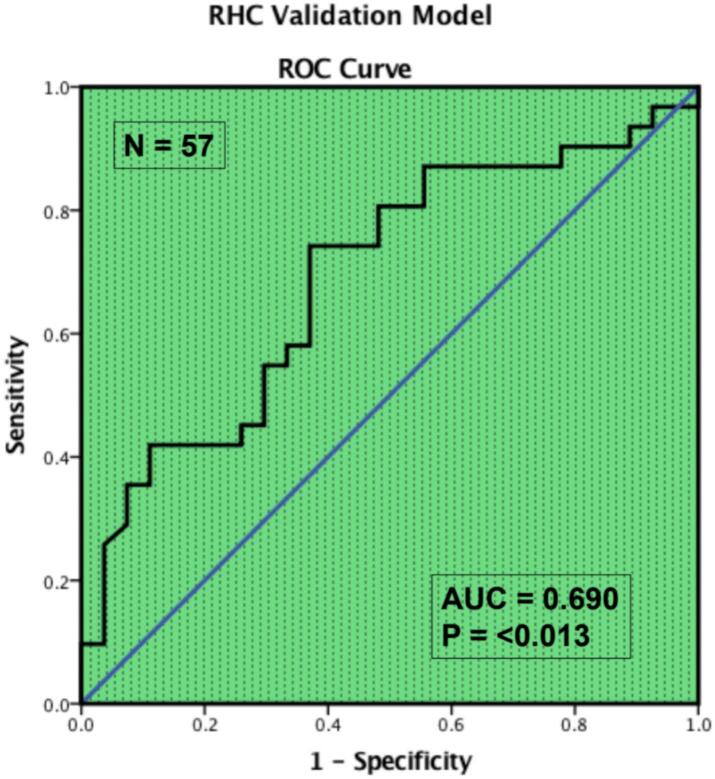
Receiver operating characteristic (ROC) curve showing the accuracy of NEDA PH-LHD prediction algorithm in identifying pulmonary hypertension due to left heart disease, compared to the gold standard right heart catheterization [20]. NEDA, National Echo Database Australia; pH-LHD, pulmonary hypertension due to left heart disease. (For interpretation of the references to colour in this figure legend, the reader is referred to the web version of this article.)

PH is most commonly due to left heart disease which can be identified using echocardiography [16]. We developed an algorithm to predict PH due to left heart disease, using only routinely acquired measurement data obtained from standard echocardiography (age, septal e’ velocity, septal E:e’ ratio, mitral inflow E:A ratio, and indexed left atrial volume). The model was developed on 151,767 echocardiograms from the National Echo Database of Australia (NEDA), validated in a further 150,979 echocardiograms and then cross-validated in 887 patients undergoing right heart catheterization which is considered ground truth for diagnosing and classifying PH [20]. As shown in Fig. 5, the algorithm showed an accuracy of approximately 70 % in identifying PH due to left heart disease. Importantly, this model does not require a tricuspid regurgitation velocity to predict PH due to LHD. However, absence of RHC data in the primary development and validation cohorts limits the model's ability to definitively distinguish PH subtypes. These findings are also derived from a conference abstract; a full peer-reviewed manuscript is not yet available, and methodological details remain limited.

A simpler model to predict PH due to left heart disease has also been developed and validated by our group, requiring only two variables: The tricuspid regurgitation peak velocity (TRV) and the septal E:e’ ratio (the echocardiographic pulmonary to left atrial ratio, or ePLAR) according to the following formula [21,22]:

ePLARm/s=TRVmsAverageE:e′ratio

**Fig. 6:**
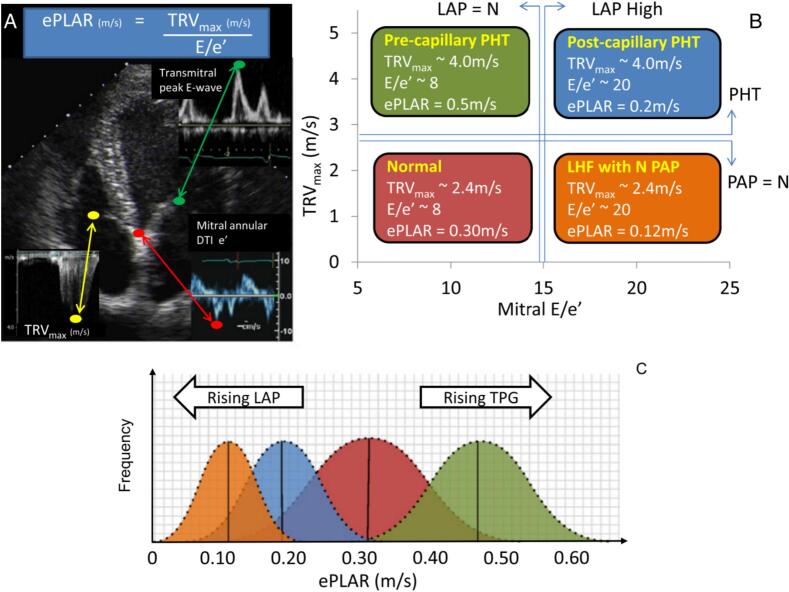
The echocardiographic pulmonary to left atrial ratio (ePLAR) explanation and example data. A. The ePLAR comprises three simple measurements: peak tricuspid regurgitation continuous wave velocity (m/s) divided by the trans-mitral peak pulsed wave Doppler *E*-wave (cm/s): peak Doppler Tissue Imaging mitral septal annular e-wave (cm/s). B. The four nominal patient subsets clinically encountered are demonstrated, with predicted bell curves displayed (C). (Image reproduced with permission from authors; Int J Cardiol 2016 Vol. 212 Pages 379–86).

This simple formula showed good capacity to differentiate pre-capillary (non-left heart) PH from post-capillary (left heart) PH in 133 PH patients classified by the gold standard (ground truth) right heart catheterization as shown in Fig. 6 [21].

#### 4.1.2 The use of artificial intelligence

**Fig. 7:**
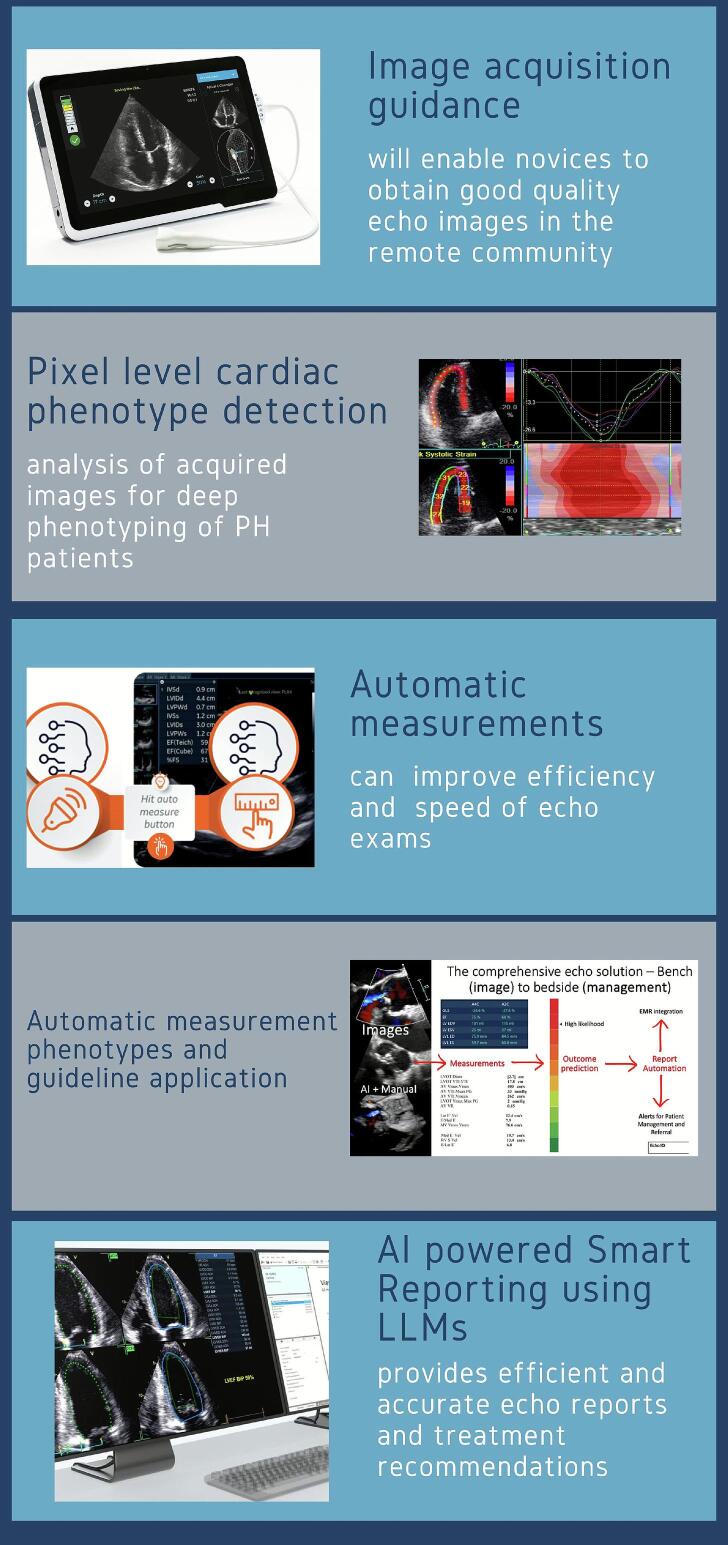
Artificial intelligence (AI) enhanced echocardiography for optimizing diagnosis of PH in isolated populations. Improved triaging of patients with the use of AI will help to inform the most appropriate imaging test for the patient. If echocardiography is appropriate, improved image quality may be obtained using AI guidance, followed by AI analysis of images for automated measurements or phenotype detection. Measurement data obtained may then be subject to further AI for application of clinical practice guidelines and risk models, and large language models (LLMs) for automation of echocardiographic reporting in preparation for review by an expert physician. AI synthesis of echocardiographic and clinical information may assist in decisions to further investigate at-risk patients with possible PH.

Incorporation of artificial intelligence (AI) into clinical echocardiography is likely to improve its efficiency, accuracy and reproducibility [23]. AI may improve the value chain of echocardiography by guiding non-expert operators such as the community health workers and nurses to obtain good quality images and hemodynamic information [24], particularly well-suited to rural, remote and inaccessible locations [25]. Diagnostic quality on-axis images obtained in this fashion can be the subject of additional AI systems to extract cardiac phenotype information (including left ventricular hypertrophy and amyloid cardiomyopathy [26]) as well as automated standard 2-dimensional and Doppler echocardiography measurements [27,28], although small, hand-held point-of-care devices have not traditionally offered pulsed-wave or continuous wave Doppler echocardiography (an important limitation to be addressed in order to improve automation) [29]. The associated automated measurements can then be the subject of further automation to diagnose diseases via incorporation of clinical practice guidelines (such as valvular heart disease or pulmonary hypertension [1,30]) or phenotype detection. Apart from on-cart applications to assist the user to obtain diagnostic quality imaging, most automation can be cloud-based and provide access to echocardiography laboratories thousands of kilometers away, to finalize expert interpretation of post-processed data. AI can also assist in echo reporting for improved efficiency using custom large language models to prepare preliminary reports prior to review by an expert clinician. Fig. 7 graphically demonstrates how such automation may be applied clinically to the screening of PH in remote, rural and/or disadvantaged populations.

**Fig. 8:**
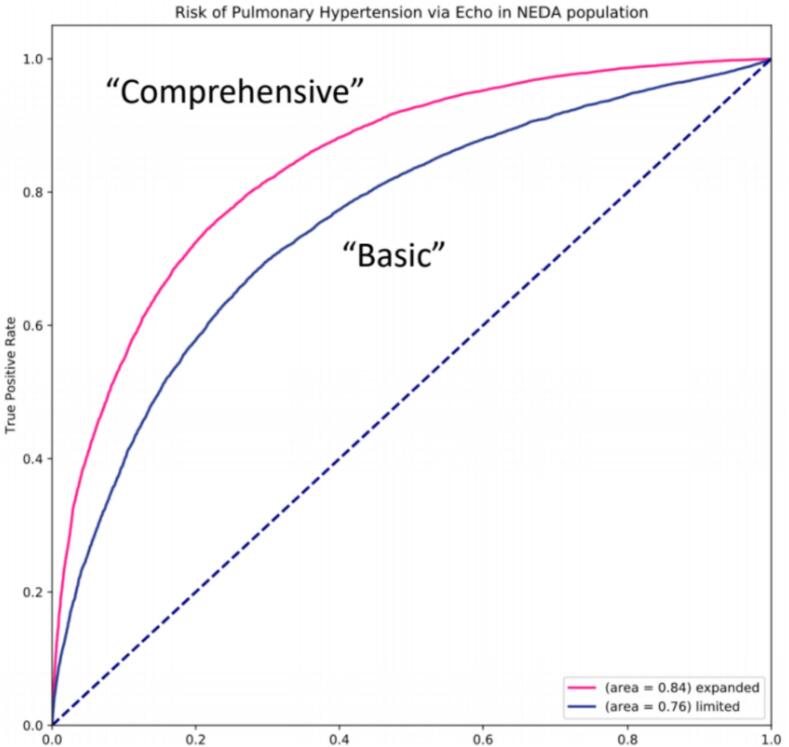
A proof-of-concept AI model trained (*n* = 371,618 studies) and tested (*n* = 31,680 studies) on NEDA data predicting pulmonary hypertension defined as TRV_max_ > 2.8 m/s using comprehensive and limited echo data. NEDA, National Echo Database of Australia. Te “comprehensive” (pink) line refers to the performance of the model when provided with all measured echocardiographic variables for each patient. The “basic” (blue) line refers to the performance of the model when provided with a limited data set comprising the typical measurements performed when measuring systolic and diastolic left ventricular function along with aortic and mitral valve velocities. The tricuspid regurgitation velocity was withheld from the model and then imputed in the prediction of PH. (For interpretation of the references to colour in this figure legend, the reader is referred to the web version of this article.)

Applying our previous demonstration that, in an unselected cohort of individuals undergoing echocardiography for clinical indications, approximately 70 % of PH has a left heart origin [6], we hypothesized that AI could automatically identify presence of PH as well as the PH phenotype using echocardiographic measurement data. We designed a proof-of-concept study using data from NEDA to first train (*n* = 371,618 studies) and then test (*n* = 31,680 studies) a deep neural network (TensorFlow, version 2.2.0) model to predict PH defined as a peak tricuspid regurgitation velocity (TRV) >2.8 m/s. The model was trained to predict the probability of all-cause PH (ground truth being TRV >2.8 m/s) by being provided all echocardiographic measurement variables except for those used to measure pulmonary artery pressure (right ventricular systolic pressure, tricuspid regurgitation velocity and gradient data and right atrial pressure), with the model penalized for incorrect predictions, followed by retraining until predictions remained constant. It is important to note that real-world echocardiography contains a varying number of echocardiographic variables depending on the clinical indication and pathology identified during the echocardiography examination. The model is capable of ingesting a variable number of input variables because of the imputation framework, which has been reported previously [31]. The trained model was then applied to the test database, never previously seen by the model. Despite PH information being withheld within the test set, the AI was able to predict the presence of PH with reasonable accuracy (AUROC curve = 0.84, *p* < 0.001). Importantly, the AI still returned a clinically meaningful PH prediction when presented with a more limited data set typically seen in screening echocardiography (such as systolic and diastolic left ventricular function and left atrial volume), with AUROC curve = 0.76, *p* < 0.001. The results of this proof-of-concept study are shown in Fig. 8. As expected from a general echocardiography model, the ground truth reflects all-cause PH rather than specific subtypes.

AI also has potential to assist in identifying individuals at risk of mortality related to PH detected through echocardiography. By harnessing machine learning and deep learning techniques, AI algorithms can be trained on large datasets of echocardiographic images, echocardiographic measurement data and/or clinical data to detect subtle patterns that might not be evident to the human eye, and outputs are free of the subjectivity typical of human interpretation. In the setting of known conditions such as PH due to left heart disease, a “ground truth” can be presented to the AI during training, and the training data set used to penalize models for incorrect classification, retraining to improve performance (usually in the order of millions of retraining cycles), and final models optimized in both sensitivity and specificity (via statistical calculation of the F1 score). While training, these algorithms can analyze the interactions of multiple parameters simultaneously, such as left and right ventricular size and systolic function, left ventricular diastolic function [32,33], the presence of valvular heart disease [[34], [35], [36]], the estimated pulmonary artery pressure (and tricuspid regurgitation data) [37,38], and other relevant factors to assess the presence and severity of pulmonary hypertension and predict mortality risk. As an example, our group has already developed AI models designed to identify the phenotype associated with severe aortic stenosis [31], showing excellent risk prediction capacity. The example provided above and in Fig. 8 demonstrates proof-of-concept using a similar design in the automated detection of the PH phenotype.

Other groups have also developed AI machine learning algorithms in diagnosing PH by echocardiography. Anand et al. demonstrated that machine learning algorithms trained on structured echocardiographic variables including chamber dimensions, Doppler indices and functional markers can effectively diagnose PH even without TRV, achieving strong diagnostic performance with an AUROC curve of 0.83; 95 % CI, 0.80 to 0.85 in a multicentre cohort consists of 7853 consecutive patients with RHC and transthoracic echocardiography performed within one week [39]. In parallel, Liao et al. developed a fully automated machine learning model that predicts pulmonary hypertension (PH) directly from parasternal short-axis echocardiographic video data, using convolutional neural networks trained and validated against right heart catheterization. In a multicentre cohort of 346 patients, the model achieved an AUROC curve of 0.945 internally and 0.950 in external validation, outperforming traditional echo metrics and demonstrating strong generalizability and operator independence [40].

Beyond echocardiography, AI has also been utilized to assist in detection of PH. Kalmady et al. developed deep learning models using over 1.6 million electrocardiograms to predict multiple cardiovascular conditions, including PH, at the population level with high diagnostic accuracy [41]. Imai et al. developed a deep learning algorithm to detect pulmonary arterial hypertension (PAH) from chest X-ray images. Trained on 418 images and tested on 101, the model produced a probability score for PAH and achieved an AUC of 0.988, with sensitivity of 0.933 and specificity of 0.982—outperforming experienced clinicians [42].

Moreover, AI can provide a more personalized approach to patient care. By analyzing echocardiographic data along with other clinical information, AI systems have the potential to stratify patients into different risk categories such as PH in the setting of lung disease, diabetes, sleep apnea, or heart failure. AI also can integrate multiple sources of clinical data, such as patient demographics, comorbidities, and laboratory results, to create comprehensive risk assessment models. These models can help identify specific patient profiles and characteristics that are indicative of a higher risk of mortality in PH, enabling healthcare providers to tailor treatment plans and interventions accordingly. Widespread use of automated PH diagnostic and risk systems is likely to improve diagnostic accuracy, efficiency and speed of diagnosis, and the ability to triage individuals at higher risk of mortality. Such an approach could lead to more timely and targeted interventions and treatment plans, potentially improving outcomes and reducing healthcare costs.

Overall, AI-powered risk assessment in PH in echocardiography shows significant promise and may be ideally suited to application where immediate access to expertise is limited. It also has the potential to enhance patient care and reduce healthcare costs by enabling more targeted and accurate risk stratification and personalized treatment strategies. Using a combination of AI models, clinicians could potentially be enhanced using AI, opening opportunities in previously inaccessible places, improved diagnosis rates of PH, and the potential to improve patient management (Table 1 and Fig. 3).

**Table 1:** A comparison of human and artificial intelligence in relation to automation in the diagnosis of pulmonary hypertension. Parallel AI systems refers to several linked AI systems, each part of the diagnostic chain in the diagnosis of PH.

	Human clinicians	Artificial intelligence
Pros	• Therapeutic relationship with patient • Empathy and compassion • Complex decision making in multi-system diseases such as PH • Capable of error-correction (e.g., measurement or input errors)	• Efficiency • No clinical, social, economic, racial bias • Less omission • No fatigue
Cons	• Potential for clinical, social, economic, racial or other bias • Fatigability • Limited availability, including geographic constraints • Time constraint • Error-prone • High cost	• High initial cost, improved cost effectiveness over time. • Depends on quality of input data • May be subject to training bias • Some systems require validation • Parallel AI systems not yet tested

### 4.2 Precise risk stratification

Accurate prognostication for PH patients is also important for risk stratification of patients and prioritization of treatments. Right heart strain using speckle tracking and three-dimensional echocardiography may be useful to detect preclinical right heart dysfunction in PH patients [43,44] and may assist with risk stratification, however these techniques, while reproducible and accurate in an advanced echo laboratory with established expertise, may not be as applicable in community settings, particularly using smaller hand-held echocardiography systems in remote and inaccessible populations. If such systems can be developed to suit this purpose, AI guided echo assessment of right ventricular-pulmonary artery (RV-PA) uncoupling would be the ideal prognostic marker in the future as RV-PA uncoupling is an important concept but difficult to grasp. RV-PA uncoupling happens when the right ventricle cannot produce enough force to match the afterload in the pulmonary artery. This results in eventual right heart failure in PH patients. Investigators in Taiwan were able to develop AI model using neural network deep learning of pairs electrocardiogram and echocardiograms to predict not only the elevated pulmonary pressure but also associated mortality [45]. This study involved 41,097 patients with paired ECG and transthoracic echocardiography within a two-week window. The model used echocardiographic pulmonary artery pressure > 50 mmHg as the reference standard and demonstrated strong diagnostic performance (AUC 0.88) and prognostic value of six-year cardiovascular mortality (HR 3.69). AI-enhanced echocardiography and electrocardiography are the potential game changers in not only diagnosing PH in early stages but also prognosticating PH patients.

### 4.3 Optimal care delivery and follow-up by dedicated pulmonary hypertension care team

To treat complex and sick patients with PH, future clinicians should arm themselves with knowledge, experience and attributes that would benefit their patients. PH clinicians should possess good communication skills to educate and guide their patients as well as to communicate with other caregivers. They should also be able to work effectively in a multidisciplinary team to deliver holistic care. These teams should include specialist physicians with interest and skills in PH diagnostics and therapeutics, specialist PH nurses and allied health team members. Primary care physicians and community health care providers should also be involved in these teams. Multidisciplinary dyspnea clinics should be set up to streamline diagnosis and management. Although advanced technologies such as AI are useful, compassion and human interaction are the two essential attributes which only human clinicians can provide (Table 1). Ongoing training and professional developments of current and future PH clinicians should not be neglected. However, AI may assist or even enhance physicians and human care givers in answering patients' questions as demonstrated in a cross-sectional study where AI generated responses to patients' questions were generally preferred by blinded health care professionals compared to physicians responses [46]. This approach may be especially useful in virtual and telehealth settings. By delegating tedious and repetitive tasks to AI, clinicians can spend more time with their patients in clinics and bedside.

AI can also improve experience for both patients and clinicians. For instance, AI based speech recognition program can provide automated generation of medical notes and correspondences which will reduce administrative burden for clinicians resulting in more clinician's time to spend with their patients. Another potential utility of AI in PH care is AI-based wearable technologies such as smartwatches assisting disease prevention as well as efficient monitoring and follow-up of PH patients [47,48].

Several highly specialized investigations may be required during evaluation of PH patients. PH clinicians should not only be familiar and competent to interpret these complex investigations but also be able to use their clinical skills and acumen to avoid unnecessary, costly, and invasive tests. They should also be aware of limitations and potential complications of current therapies and interventions and able to guide their patients. Treatment should be individualized based on the background and expectations of each individual patient. Patients and their families should be well informed about their conditions and available treatments to enable shared decision making.

## 5 Conclusion

Despite improved understanding of PH and technological advances in diagnosis and treatment, PH patients are still facing significant morbidity and mortality. The problem is compounded by massive disease burden, heterogeneity of PH and lack of resources especially in the remote regions. We have outlined the recent advances in PH diagnosis and management in this review and advocated personalized precise treatment for every PH patient with the aid of modern technologies.

## CRediT authorship contribution statement

**Pyi Naing:** Writing – review & editing, Writing – original draft, Visualization, Conceptualization. **Gregory M. Scalia:** Writing – review & editing, Supervision. **Dale Murdoch:** Writing – review & editing. **Isuru Ranasinghe:** Writing – review & editing. **Doug L. Forrester:** Writing – review & editing, Supervision. **Geoff Strange:** Writing – review & editing, Supervision, Conceptualization. **David Playford:** Writing – review & editing, Supervision, Conceptualization.

## Declaration of competing interest

Dr. Pyi Naing received an Australian Government Research Training Program (RTP) Scholarship to support his PhD studies. We did not seek ethical clearance for this review paper.
